# What are the benefits of cognitive enhancers for Alzheimer's Disease: use of Population Impact Measures

**DOI:** 10.1186/1471-2318-7-25

**Published:** 2007-11-06

**Authors:** Udaya P Gammanpila, Alistair Burns, Richard F Heller, Nitin Purandare

**Affiliations:** 1 Specialist Registrar in Psychiatry, Forest House, Royal Oldham Hospital, Rochdale Road, Oldham, OL1 2JH, UK; 2 Professor of Old Age Psychiatry, University of Manchester, Education and Research Centre, Manchester, M23 9LT, UK; 3 Emeritus Professor, University of Manchester: 144/10 Commercial Street, Edinburgh EH6 6LB, UK; 4 Senior Lecturer in Old Age Psychiatry, University of Manchester, Education and Research Centre, Manchester, M23 9LT, UK

## Abstract

**Background:**

The study aims to quantify the population impact of prescribing cholinesterase inhibitors to slow the cognitive decline in Alzheimer's disease (AD), and to compare with the benefit of treating hypertension to prevent the onset of AD.

**Methods:**

Literature review to ascertain the prevalence of AD, benefits of interventions, analysis of local and national surveys to measure the current use of interventions in the relevant population and application of the relevant findings to calculate Population Impact Measures. The Number of Events Prevented in a Population (NEPP) by the intervention over a defined time period is calculated for a UK urban population in one Local Authority (population size 217,000).

**Results:**

Treatment of all eligible patients with mild to moderate AD with Cholinesterase Inhibitors would prevent cognitive deterioration (measured by ADAS – cog scale) in 123.6 (95% Confidence Intervals (CI) 82.3, 169.1), 16.4 (95% CI 2.1, 31.2) would show a mild improvement (4 points or more on the ADAS – cog scale) and 2.6 (95% CI 0.2, 5.8) would show an improvement of 7 points or more over a period of 6 months. This would require the treatment of 406 patients with Cholinesterase Inhibitors.

Increasing from the current treatment rate of 46% of eligible patients to 'best practice' level would prevent cognitive deterioration in 66.8 (95% CI 44.0, 92.6), 8.99 (95% CI 1.2, 16.8) and 1.4 (95% CI 0.11, 3.2) would improve by 4 and 7 points respectively on the ADAS – cog scale over 6 months. This would require the treatment of an extra 187 patients with Cholinesterase Inhibitors beyond current practice, at an additional annual direct drug cost of £187,000.

Improving the treatment of hypertension from current practice by 20% could prevent 8.2 (95% CI 2.3, 16.8) incident cases of AD in the next year. This would require the treatment of an extra 2711 patients with antihypertensive drugs.

**Conclusion:**

Population Impact Measures are a new method to allow a demonstration of the magnitude of the benefit for the whole population following interventions. The use of drugs to slow cognitive decline, or to prevent AD by treating hypertension, can thus be assessed in a prioritisation exercise in competition with alternative use of resources.

## Background

There are a number of interventions that have been found to be effective or that have the potential of reducing the burden of Alzheimer's disease (AD). These can be classified according to whether the intervention is aimed at the general population (to prevent the onset), or at patients with AD (to improve or slow the progression of cognitive decline). These interventions include lowering blood pressure and cholesterol levels and the use of hormone replacement, vitamins, non-steroidal anti-inflammatory drugs to prevent the onset of AD and the use of Cholinesterase Inhibitors (ChEIs) and cognitive behavioural interventions to slow the cognitive decline. While those with mild cognitive impairment are a high risk group for the development of AD [1], and although no specific interventions have been shown to be of benefit, they should also benefit from the interventions that help the general population. This therefore has a large potential impact in view of the high risk that this population runs of developing AD.

Of the potential preventive measures the best evidence, from randomised control trials, is for treating hypertension [2,3], although there is debate about this [4]. Similarly ChEIs have the best evidence for cognitive enhancement [5,6]. Hence, we chose these over other potential preventive and treatment interventions [7-12].

We have developed new measures of the population impact of interventions, which estimate the numbers of people in a defined population who will benefit from an intervention [13-18], and are population extensions of the clinically relevant number needed to treat (NNT). The measures require an estimate of the risk of the health outcome we are examining, the benefits of the proposed intervention derived from the literature, how common is the condition and the current use of the intervention in the population. These measures of health improvement can be set against the cost of each intervention and appropriate priorities could be established that will maximise gain for the population. As examples of this, we have shown that interventions for schizophrenia, going from current to best practice in a population of 100,000, would prevent between 6 and 40 hospitalizations and between 6 and 44 relapses in a year. Similarly, depression interventions would lead to between 100 and 325 relapses prevented [18,19]. After acute myocardial infarction, increasing the use of beta-blockers would prevent 11 deaths in the next year among a population of 100,000 at a cost of £158 per life-year saved, compared with 4 deaths prevented at a cost of £423 per life saved by increasing the smoking quit rate [17].

By comparing the population benefit of different interventions, and their costs, it will be possible to make prioritisation decisions about how to make the biggest impact on reducing the disease burden of AD on the population, and to make comparisons with the impact of interventions for other conditions. This will be of value to policy makers and consumers as well as to those at risk of suffering from AD.

This study thus has aimed to quantify the population impact of prescribing ChEIs, and compare with the treatment of hypertension, on AD in a local population.

## Methods

The study was located in Oldham, a borough in NW England with a population of 217,000 people. Its aged care services include two sector based old-age psychiatry community mental health team with two consultant psychiatrists. The community psychiatry teams and the social services are well integrated to provide comprehensive care. Oldham was selected as a typical urban borough with availability of the required data from local surveys. Ethics approval was not sought as the paper relied on review of the literature and no new data were collected.

Step 1 – literature review to examine key interventions with established or potential benefit for AD, among the general population, among those with mild cognitive decline and those with AD. We focused on the relative risk reduction and assumed that they can be generalised to different populations [20]. The main outcome measure was taken as cognitive decline, as this is an important outcome in AD and one for which measures of Relative Risk Reduction are available.

Step 2 – we examined the current practice of the use of preventive and treatment agents, where possible from the relevant population (Oldham). This included a retrospective audit on prescription of ChEIs, analysing the case notes and pharmacy prescriptions from 2002 to 2005 conducted in Oldham and other health areas by the University of Manchester psychiatry division co-ordinated by one of us (NP) [21]. For hypertension, national figures for proportions of the population being treated were used, as published in the Health Survey for England [22]. These allow an examination of the change from current to 'best practice', defined as the proportion of people with the condition who should be treated under ideal conditions, adjusted for adherence to the therapy.

Step 3 – identify the population characteristics of Oldham to identify the age structure. This allowed us to make estimates from real populations to which the results can be applied.

Step 4 – identify costs of different interventions. We used estimates of drug costs where possible but did not make a more detailed estimate of the costs of the different interventions.

Step 5 – calculation of Population Impact Measures. This involved application of the Relative Risk Reductions, to the population we have identified, of both the total impact amongst those with mild to moderate AD as well as the change from current use of interventions. The measure we used in this study was the Number of Events Prevented in a Population (NEPP) which describes the impact of the interventions and is defined as "the number of events prevented by the intervention in your population over a defined time period [15,19]." The Number of Events Prevented in the Population (NEPP) is calculated as follows:

NEPP = n*P_d_*P_e_*r_u_*RRR

where

n = population size

P_d _= the prevalence of the disease in the population

P_e _= the proportion eligible for treatment

r_u _= the risk of event of interest in the untreated group or base line risk

RRR = the relative risk reduction associated with the treatment.

In order to reflect the incremental effect of changing from current to 'best practice' and to adjust for levels of compliance, the proportion eligible for treatment, P_e_, is (P_b _- P_t_)P_c_, where P_t _is the proportion currently treated, P_b _is the proportion that would be treated if best practice was adopted and P_c _is the proportion of the population who are compliant with (adherent to) their medication.

Calculations of the NEPP for different outcomes were calculated according to the above formula, using an on-line calculator [23]. This provides 95% Confidence Intervals using a simulation method with 10,000 iterations. The calculation requires the population sizes from which the various estimates in the formula are derived, and these were taken from the sources used to derive the estimates themselves. In addition, the Confidence intervals for the Relative Risk Reductions are required, and these were re-calculated from the original trial data in the relevant publications, using an on-line calculator [24].

### Cholinesterase inhibitors (ChEI's)

The population of 31600 aged 65 or above for Oldham was obtained, to reflect those at high risk of developing AD and at whom services are targeted, by population projections for 2006 from census data (Simpson and Gavalas, Cathie Marsh Centre for Census and Survey Research, internal publication).

The prevalence of AD was obtained from global consensus data for dementia ≥60 years of 5.4%, and an estimate that 50–70% of those with dementia have AD, giving an assumption that 3% of the population have AD (61% of those with dementia) [25,26].

Memory assessment and treatment services (MATS) at Oldham followed the 2001 NICE guidelines on prescribing ChEI's which recommend that patients with mild to moderate degree of AD are eligible for treatment [5] (this changed by the time of the 2006 recommendation, which limited treatment recommendation to those with moderate dementia) [6]. Disease severity was based on the mini mental state examination (MMSE) score which classifies mild 21–26, moderate 10–20, moderately severe 10–14 and severe AD with a score less than 10. About 50–64% of the patients suffer from mild to moderate degree [5]. From these figures, the proportion of the AD population eligible for treatment was taken to be 0.55. Generally, very few patients with AD have absolute contra-indications for ChEIs and the local audit [21] found 78% of those treated were still on treatment at 6 months (the figure we have used for adherence to treatment). A similar figure of 82% adherence was seen elsewhere [27].

The Relative Risk Reduction of cognitive decline by treatment with ChEI's and the risk of event of interest in the untreated (placebo) group or the base line risk was taken from Livingston and Katona [28], and re-calculated from the original trial [29]. The confidence intervals around the Relative Risk Reduction were calculated from the original trial data [29], using an on-line calculator [24].

### Treatment of hypertension

The systolic hypertension in Europe study (Syst-Eur) reported a 55% Relative Risk Reduction (95% Confidence Intervals 24%, 73%) in dementia associated with treatment in an extended follow-up study of 3.9 years [3]. Eligible patients for the study had no dementia, were at least 60 yeas old (median 68) and had a sitting systolic blood pressure ranging from 160 to 219 mmHg and the diastolic blood pressure was below 95 mmHg. In order to assess the outcome of AD alone, the original data in the paper were used to calculate the Relative Risk Reduction and 95% Confidence Intervals as for ChEIs above.

The health survey of England 2003 [22,17] shows that 52% of men and 56% of women aged 65–74 as well as 71% of men and 65% of women aged 75 or more have high blood pressure above 160/95. We took 55% as a characteristic average of the above figures as the prevalence of hypertension at 160/95 in the reference population. The Health Survey of England 2003 also shows that at a cut-off point of 160/95, 11.9% of men aged 65–74 and 23.9% of men aged 75 and more have either treated hypertension (i.e. on treatment but still hypertensive) or untreated hypertension. For women the respective figures are 18% and 27.4%. We took 20% as a rough mean of the above figures as the proportion who could benefit from improved treatment, and took 78% compliance with treatment to be consistent with the data for cognitive enhancers above. Baseline risk of the development of AD was taken from the control group of the randomised controlled trial [3].

## Results

### Cholinesterase inhibitors

Table 1 shows the estimates and the results of the calculations. If all the eligible patients in the population were to be treated, (with compliance taken into account) we could expect that it would prevent cognitive deterioration (as measured on ADAS cog scale) in 123.6 (95% Confidence Intervals (CI) 82.3, 169.1), 16.4 (95% CI 2.1, 31.2) would show a mild improvement (4 points or more on the ADAS – cog scale) and 2.6 (95% CI 0.2, 5.8) would show an improvement of 7 points or more over a period of 6 months. This would require the treatment of 406 patients with ChEIs.

**Table 1:** Calculation of Number of events prevented in the population (NEPP). Number in the population is 31600, and adherence with therapy 78% in each group.

Outcome achieved	Prevalence of the disease in the population (P_d_)	Proportion eligible for treatment (P_e_)	Number given treatment	Risk of event of interest in the untreated group (r_u_)	Relative Risk Reduction (95% CI)	Number of events prevented in the population** (95% CI)
Cognitive enhancers to all eligible with compliance taken into account						

No deterioration*	0.03	0.55	406	0.577	0.527 (0.321, 0.671)	123.6 (82.3, 169.1)
4 point improvement*	0.03	0.55	406	0.268	0.151 (0.01, 0.272)	16.4 (2.1, 31.2)
7 point improvement*	0.03	0.55	406	0.078	0.082 (0.004, 0.154)	2.6 (0.2, 5.8)

Cognitive enhancers to those eligible but not currently treated						

No deterioration*	0.03	0.253	187	0.577	0.527 (0.321, 0.671)	66.8 (44.0, 92.6)
4 point improvement*	0.03	0.253	187	0.268	0.151 (0.01, 0.272)	8.9 (1.2, 16.8)
7 point improvement*	0.03	0.253	187	0.078	0.082 (0.004, 0.154)	1.4 (0.11, 3.2)

Treatment of hypertension in the population for those eligible but not currently treated						

Development of AD	0.55	0.20	2711	0.005	0.605 (0.229, 0.798)	8.2 (2.3, 16.8)

*in ADAS-cog scale

**applies to 6-month outcome for cognitive enhancers and 1-year for hypertension treatment

To assess the benefit of going from current to 'best practice', we used unpublished results from the local audit data [21] showing that 46% of the eligible patients were treated with ChEI's (although this may well be an over-estimate [30]). If we are to apply these findings in an incremental manner from current practice to the best practice, i.e. the rest of the eligible patients were to be treated, we could expect an additional benefit of preventing cognitive deterioration in 66.8 (95% CI 44.0, 92.6), 8.9 (95% CI 1.2, 16.8) showing a mild improvement and 1.4 (95% CI 0.11, 3.2) would improve by 7 points on the ADAS-cog scale. This would require the treatment of an extra 187 patients with ChEIs beyond current practice.

The annual drug cost for a patient to be treated with ChEI's is taken as approximately £1,000 per year [5]. Since going from current to 'best practice' would involve the treatment of an extra 187 patients, the direct additional cost for drugs at Oldham would be about £187,000 per year. In addition the MATS clinic at Oldham takes one-fifth of the consultants' time, the nursing services and the costs for routine investigations for each patient at the beginning of treatment, and these costs would be increased if the additional patients were to be treated. In addition, there would be costs associated with the detection of these extra patients and their recruitment to the clinic and their on-going care.

#### Treatment of Hypertension

Table 1 shows that increasing the current treatment rates for hypertension by 20%, would lead to the prevention of 8.2 (95% CI 2.3, 16.8) incident cases of AD in the next year. This would require the treatment of an extra 2711 patients with antihypertensive drugs.

## Discussion

We have used Population Impact Measures to show the benefits to a whole population of interventions aimed at either slowing cognitive decline, or averting new cases, of those with AD. The data are presented in terms of absolute numbers amongst the whole elderly population of an area such as Oldham in North West England. We have also examined the impact of change from current to 'best practice', defined as the proportion of people with the condition who should be treated under ideal conditions, adjusted for a realistic assessment of adherence to the therapy. As well as the numbers of outcome events prevented, we present the numbers of people who would need to be treated to achieve these outcomes. The use of cognitive enhancers to treat those already diagnosed with AD is contrasted with treatment of hypertension in the community to prevent the onset of AD, although both outcomes are of potential value to the population. The combination of different interventions could also be investigated using this methodology, although it would depend on the existence of original trial data to provide estimates of the benefit.

Our methods take into account the local prevalence of the disease, adherence to the interventions, current practice and the services available. The prevalence estimates we have made are from the literature, although local data from the population in question would be preferable. Our use of local audit data is an example of the value of local data, although a study in a different population found only 5% of eligible patients to be treated with ChEI's [30] in contrast to our 46%.

In a cost-effectiveness analysis, such as that used by NICE [5,6], value weights are assigned to each outcome, whereas we suggest that the prioritisation in 'competition' with other demands on resources is performed by the policymaker on the basis of the impact on the population – we have termed this 'population cost-impact analysis' [17]. We have not produced full costing estimates, but have indicated the kind of data that would be required – such costs are likely to differ according to local settings. Our conclusions shed a different light to those of NICE clinical guidelines, although and part of this difference may be due to choice of outcome measure. We have used cognitive decline, not nursing home admission.

The calculation of population impact is a relatively new and developing area of research [31]. Like other population measures, our calculations may be criticised for being crude measures. Their accuracy is dependent on the accuracy of the data on individual variables in the equation.

For example, it is difficult to set an exact cost and benefit in relation to cognitive functioning due to the lack of consensus in this area as shown by the difficulties which arose in the consultations leading to the recent NICE guideline review [6]. Studies do show that the cost of care is more with the worsening of cognitive disability [32], including a UK study which assessed the cost of care for non-institutionalised patients with AD over a 3 month period showing the significance of cost variation according to the severity of cognitive disability [33]. The total mean cost for control subjects was £387, mild disability £6,616, moderate £10,250 and severe £13,593 and more importantly indirect cost as mainly the time spent by the care givers was calculated as the main cost component in all groups 68.6% followed by medical costs 24.7%. Our calculations of the population impact of actual treatment for a specific population need to be put in the context of the estimated cost of dementia for that population.

In estimating the cost we have only used the rough estimate of drug costs where possible, in addition the local service planners could take into account the actual cost involved in providing the services such as cost of investigations and for the staff, and in particular the on-going costs of long-term treatment and clinical care.

Our findings could be further supplemented by a local qualitative study analysing the views of patients, carers and clinicians regarding the benefits of interventions in AD.

The relative risk reduction figures used for ChEI's are from a study of a follow-up period of only 24 weeks [29]. There are a number of other studies of the benefits of these drugs, including a Cochrane Review [34], which confirm a benefit in terms of reduction of cognitive decline. We have chosen to make the calculations here based on one randomised controlled trial, due to the presentation of outcome by degrees of cognitive change and the ability to incorporate estimates of relative Risk Reduction which are necessary for the calculation of NEPP.

The impact of treatment of hypertension on AD is more debatable. We have used long-term follow up from the Systolic Hypertension in Europe study [3], which builds on an earlier report of benefit [2], despite the fact that a later meta-analysis [4] showed a much smaller and non-significant effect. The meta-analysis concluded that there were a number of methodological problems limiting the conclusions such as the pharmacodynamic properties of the anti-hypertensive used and the increasing use of antihypertensives over time in the control group, and suggested further analyses. In the absence of a clear consensus at this time, the data we present should be regarded as indicative of the impact that treatment of hypertension may have on the development of AD, and the potential value of the kind of calculations we have made. We have made a number of other assumptions from the data reported in the trial, where the patients had elevated systolic blood pressure between 160–219 mm hg with diastolic blood pressure below 95 but the prevalence figures we used are for blood pressure above 160/95 as the figures specifically for the above category were not available. We have made our own calculations of the Relative Risk Reduction and 95% Confidence Intervals, from the raw published data, for the outcome of AD, as these are only reported for all causes of dementia [3]

## Conclusion

Population Impact Measures are a new method to allow a demonstration of the magnitude of the benefit for the whole population following interventions. The use of drugs to slow cognitive decline, or to prevent AD by treating hypertension, can thus be assessed in a prioritisation exercise in competition with alternative use of resources.

## Competing interests

The author(s) declare that they have no competing interests.

## Authors' contributions

UPG and RFH designed the study, in consultation with AB and NP. UPG collected the data and performed the analyses. All authors discussed and interpreted the results. UPG wrote the first draft of the paper with RFH, and all authors commented on and revised the draft and have read and approved the final manuscript.

## Pre-publication history

The pre-publication history for this paper can be accessed here:


